# Physical Activity and Its Determinants among Senior Residents of Podlasie, a Green Region of Poland, Based on the National PolSenior Study

**DOI:** 10.3390/ijerph182010816

**Published:** 2021-10-14

**Authors:** Rafał Rowiński, Grażyna Kowalska, Mariusz Kozakiewicz, Kornelia Kędziora-Kornatowska, Maciej Kornatowski, Joanna Hawlena, Karolina Rowińska

**Affiliations:** 1 Department of Tourism and Recreation, University of Life Sciences, Akademicka Street 15, 20-950 Lublin, Poland; badaniaawf@wp.pl (R.R.); hawlena@interia.pl (J.H.); 2 Department of Socio Economic Activity, Institute of Socio-Economic Activity, 01-802 Warsaw, Poland; 3 Department of Food Chemistry, Nicolaus Copernicus University Collegium Medicum, 85-626 Bydgoszcz, Poland; markoz@cm.umk.pl; 4 Department of Geriatrics, Faculty of Health Sciences, Nicolaus Copernicus University of Toruń, 85-626 Toruń, Poland; kornelia.kornatowska@cm.umk.pl; 5 Department of Geriatrics, Faculty of Health Sciences, L. Rydgier Collegium Medicum, 85-626 Bydgoszcz, Poland; 6 Faculty of Health Sciences, Mazovian Public College, 09-402 Płock, Poland; kikgeriat@cm.umk.pl; 7 Department of Movement Teaching, Józef Piłsudski University of Physical Education, 00-968 Warsaw, Poland; karolina.rowinska@awf.edu.pl

**Keywords:** physical activity, older people, aging, healthy aging

## Abstract

Physical activity is essential at all stages of life, and particularly so in the later years. The main objectives of the present work was to evaluate the physical activity of seniors, aged 65 years and older, living in the area of the Podlaskie voivodeship (Podlasie), Poland, based on the results of the nationwide PolSenior project, and to formulate recommendations for health policies implemented by both national and local government units. The study was performed as part of the national PolSenior project, whose aim was to evaluate various aspects of aging in Poland. The physical activity of a group of 186 randomly-selected people aged 65 years and above, 94 men and 92 women, was evaluated by questionnaire. The analysis included participants who took part in physical activity at least several times a week. Although all participants reported a decline in physical activity with age, the men remained physically active for longer. Among the respondents, 68.3% of women and 62.7% of men took short walks around the house as the main form of exercise, with working on the allotment or garden being another frequent activity; however, this was more common among men (53.9%) than women (34.7%). In addition, men were nearly twice as likely to take part in cycling (31.5%) than women (13.1%). The greatest motivation for physical activity given by the respondents was health, as noted by 73.8% of the men and 77.7% of the women. The physical activity of seniors in Podlasie is unsatisfactory and does not fulfil the WHO recommendations regarding the prophylaxis and prevention of chronic illness. This level does not, however, significantly differ from that reported in the nationwide PolSenior study or in other European countries in the Eurobarometer study. The decline in physical activity with age highlights the need for its greater promotion among seniors by local authorities. Such initiatives will help maintain the physical fitness and independence of this age group and contribute to a greater quality of life.

## 1. Introduction

In Poland, as in other countries with varied societies and economies, both in Europe and beyond, physical activity (PA) remains at an unsatisfactory level among seniors [1].

A study of participation in recreational activity and sport by the Polish Central Statistical Office (GUS) in 2008 found that 79.3% of the population aged 60 years and above was not physically active. The situation was little better in younger age groups: it was found that almost three quarters (73.4%) of those aged 50–59 years were physically inactive, as well as 67.5% of those aged 40–49 years, and more than half of those (59.6%) aged 30–39 years [2]. Older people, aged over 65 years, were the least physically active of all the groups [3,4]. Participation in Leisure Time Physical Activity (LTPA) is influenced by a range of socio-demographic, personal, behavioral, and psychological factors, as well as various environmental influences [5,6,7,8]. In addition, a literature analysis suggests that age, sex, education, occupation and place of residence can also play a role. LTPA is more often engaged in by people with higher education, those living in large cities, and those working professionally, as non-blue collar workers [9,10,11,12,13,14,15,16,17].

Such socio-demographic determinants should be taken into consideration by local government when forming PA policies aimed at including seniors.

It should be remembered that PA is necessary for the proper development and functioning of a person at all stages of life, particularly during old age. A low level of PA leads to a fall in physical fitness to below norms. It is accepted that physical fitness gradually decreases with age during adulthood [18]; however, physically active people can maintain their physical fitness level within the age norm by undertaking regular activity at a level above their daily functional needs. Despite this, a decline in physical fitness above the norm, usually occurring in inactive people, can lead to the elderly experiencing great difficulties in coping with utilitarian domestic and self-care activities; this can hamper their own ability to meet their needs and incur greater state expenditure on medical care and social assistance. For seniors, maintaining an appropriate level of physical fitness allows them to retain their independence and the possibility of self-fulfillment [1,17].

As they age, those who do not take part in regular PA demonstrate a greater decline in physical fitness than those who participate in regular physical activity at a higher level than normal daily activities. They also demonstrate a decline in physical efficiency, one of the features of the body’s motor skills, which shows the body’s ability to undertake long physical efforts without rapidly becoming tired. Sedentary people demonstrate a faster rate of decline in exercise capacity with age, amounting to about 10% per decade of life, compared to around 5% in the case of very physically active people [19,20].

A similar trend can be seen for muscle mass and, hence, muscle strength. It has been found that 80-year-olds typically demonstrate 30–50% less muscle mass than 40-year-olds. [18,19,20,21,22]. This decline in muscle strength, particularly in the elderly, can also contribute to a loss of independence [23,24]. One reason for the decline in exercise capacity with age is, apart from chronic diseases, a sedentary lifestyle [25,26].

Although skeletal muscle mass accounts for about 45% of the body weight in young people, it decreases to about 27% in those over 70 years of age [27]. This decline in strength is also typically accompanied by a decrease in flexibility, as well as physical fitness, which determines exercise capacity. Agility and motor coordination also deteriorate. Regular PA may slow the loss of muscle strength, thus improving functional performance and reducing the risk of falls in the elderly [28,29,30].

The beneficial effects of regular PA among the elderly are manifested as reductions in the risk of premature death, coronary artery disease, stroke and arterial hypertension, as well as type II diabetes, metabolic syndrome, colon and breast cancer [31]. Systematic exercise also reduces body weight, improves cardiovascular, muscular and cognitive performance, and reduces the risk of depression [29,30,31,32].

The 2020 WHO guidelines indicate that PA plays an important role in the prevention of chronic diseases; however, people over 65 years and those with disabilities or chronic diseases should be careful and consult a doctor before exercising. In the elderly, PA helps to maintain good bone health and overall body function. As part of their weekly physical activity, older people should engage in a variety of multi-component physical exercises emphasizing balance, and in strength training of moderate or greater intensity. These exercises should be performed three or more days a week to increase exercise capacity and prevent falls. The 2020 WHO guidelines, updating those issued in 2010, should be implemented into national health policies in line with the WHO Global Action Plan on Physical Activity 2018–2030 [33].

PA is considered as a factor contributing to health and occupies a significant place in the health policy of Poland. Policies aimed at promoting healthy and active aging through physical activity at the national level were defined by the National Health Program implemented in 2016–2020, which is to be continued in the following years. The National Health Program is a strategic document for public health, and, therefore, the basis for activities in this area. Pursuant to Art. 9 of the Act of 11 September 2015 on public health, the NHP is drawn up for a period not shorter than 5 years [34,35].

The strategic aim of the Program was to extend the healthy life of Poles, improve health-related quality of life and reduce social inequalities in health. The implementation of the program was to be carried out by local authority units. The budget of the program exceeded PLN 210 million [34].

Podlasie, currently the Podlaskie voivodeship, is one of the 16 local government units. This is the green region of Poland, with a forest cover of 30.9%: the mean afforestation for the whole of Poland being 30.34%. It is situated in the north-eastern part of the country, located at the geographical center of Europe. It is the voivodeship with the largest number of national parks, with four in the area: Białowieża National Park, Biebrza National Park, Narew National Park and Wigry National Park. This is far higher than the average for Poland, being 1.43 per voivodship. Podlasie is also interesting because of its international neighborhood: its position in the north-eastern corner of Poland borders ex-Soviet Belarus to the east, and Lithuania, a European Union member, to the north-east [36].

The population of the voivodeship aged 65 and above comprised over 176,000 people: 108 thousand women and 67 thousand men. More than half of the women aged 65 and above (53%) live in cities, and 47% in villages; in turn, 51% of the men live in cities, and 49% in rural areas [36]. The mean life expectancy in Podlasie was representative of the rest of the country, i.e., 73.3 years for men and 81.8 years for women, compared to 72.4 years for men and 80.9 years for women in Poland as a whole. High male mortality can also be seen both in the Podlaskie voivodeship and throughout Poland. In the analyzed year, the life expectancy of men and women in Podlasie differed by 8.5 years, both nationwide and in Podlasie. Compared to the beginning of the 1990s, the life expectancy of men had increased by 6.2 years and that of women by 5.0 years in Podlasie, and by 6.2 and 5.7 years, respectively, nationwide [37].

The detailed aims of the study were to evaluate engagement in LTPA and investigate its determinants among seniors aged 65 and above living in Podlasie, based on the results of the nationwide PolSenior project. It also aims to formulate recommendations for health policies implemented by both national and local government. In the presented work, particular attention is paid to the formulation of health policy aimed at seniors living in the Podlaskie voivodeship, which is shaped by local government. However, to formulate effective programs for increasing participation in PA in any social groups, it is first necessary to know its determinants [10]. A hypothesis was adopted that there are differences between the variables: age, sex, size of the place of residence, socio-occupational status and types of LTPA.

## 2. Materials and Methods

Results presented in this paper are part of the research conducted between 2008 and 2012 within the framework of the national project PolSenior, whose aim was to assess various aspects of aging among people in Poland, including the health, social and economic situation of seniors over 65 years of age. PolSenior is the first multidisciplinary and multifaceted research project of such size to be performed on aging in Poland.

The project was coordinated by the International Institute of Molecular and Cell Biology in Warsaw. The ethical consent for the research was given by the Bioethics Committee at the Medical University of Silesia in Katowice. The participants for the nationwide study were selected randomly in a three-stage, tiered scheme; all participants received a clinical examination and demographic survey [38,39].

The selection scheme was performed as a three-stage draw to obtain a nationwide, representative group of respondents. In the first stage, random locations (districts) were chosen throughout the country. In the second stage, in the selected districts, streets were selected in the urban districts and urban–rural districts, or villages in the more rural parts. The urban areas of the centers were divided into five groups depending on the number of inhabitants: up to 20,000 inhabitants; from 20,001 to 50,000 inhabitants; from 50,001 to 200,000 inhabitants; from 200,001 to 500,000 inhabitants; over 500,000 inhabitants. Finally, in the third stage, specific respondents were selected at random from the streets and villages in stage two; this was performed by the Ministry of the Interior and Administration on the basis of PESEL number: this being a unique number for each Polish citizen with an ID card [38,39].

In total, 5695 respondents took part in the PolSenior survey. Of these, 5516 respondents received a medical examination performed by an appropriately-trained nurse [38,39] and a social interview; this number constituted 35% of the randomly-selected addresses. By far the most common reason for non-participation was refusal to participate by the recipient (32% of all addresses) or by those living with them (6%). In total, the survey could not be completed at 49% (7681) of the valid randomly-selected addresses, and a further 14% of the addresses were invalid, i.e., the randomly-selected person could not be found, due to death, moving or absence for the duration of the study. In the Podlaskie voivodeship, the sample implementation effectiveness index, i.e., the relationship between the number of addresses where the survey was conducted to the number of all correct addresses, was 53%, compared to 42.58% for the nationwide sample [40].

Our findings present the physical activity and factors influencing PA participation of the inhabitants of Podlasie, the “green” region of Poland. We looked for differences and relationships between such variables as: age, sex, size place of residence, and socio-professional group and type of LTPA such as: short walks near the house, walking or hiking lasting a few hours, exercise such as gymnastics, aerobics, cycling and working in garden. In the light of the terminology of American sociology, we adopted non-blue collar workers as employees who do not undertake manual work and require physical effort. Blue collar workers are those who undertake manual work and those that require physical effort.

We also looked for differences and dependencies between variables such as: age, sex, size of the place of residence, socio-professional group and motives for LTPA, such as: for health reasons, to relax, to “kill time, out of habit”, “I exercised when I was young” and “on doctor’s advice”.

The respondents were asked about the forms of their PA as part of moderate LTPA in the last 12 months; they were also asked about the their motivation for taking part in PA. The survey addressed the following points:

- What form of activity do the surveyed seniors take part in?
- Are there any differences in PA, and if so, do they depend on sex, age, socio-economic position and place of residence (size of city)?
- Why do the surveyed seniors undertake PA?

In this particular voivodeship, a total of 186 elderly people aged 65 and above were examined: 94 men and 92 women. To be eligible for inclusion in the study, the participants had to take part in LTPA several times a week or more (not less than 2 times a week and not more than 7 times a week, i.e., every day). Of these, 178 people, 89 men and 89 women, reported having a professional occupation; no data was available for five men and three women. Among the respondents participating in LTPA, none reported cognitive impairment or the need for support from others in daily functioning due to a disability. The results were analyzed with regard to age group, divided into 65–74 years, 75–84 years and over 85 years, as well as the type of place of residence and the professional status of the respondents. The numbers of the respondents in each of these divisions are presented in Table 1.

Our sociological study did not take into account physiological parameters. The statistical relationships between age, sex, size of the place of residence, socio-professional status and types of LTPA were analysed using the Chi-squared test. Any differences between means at a *p*-value less than 0.05 were considered statistically significant.

## 3. Results

Our results indicate that participation in long walks and hikes lasting a number of hours, gymnastics, cycling, working on an allotment or in the garden decreases with age (Table 1). Among women aged 75–84 and 85 and older, no participation in long walks was recorded. A visible trend of decreasing participation in PA with age was also observed among men, but it did not fall below 4% in those aged 85 and above. The exception are short walks around the house, where participation by men remains greater than 60%, regardless of age. Interestingly, significantly higher proportions of men took part in cycling than women in the 75–84 (31.4% compared to 4%) and over 85 age groups (60.1% compared to 37.1%) (Table 1).

Although gymnastics is a form of LTPA with a high pro-health value, participation was lower than in other physical activities, such as short walks around the house, longer walks near the place of residence, cycling or working on the allotment or in the garden. Women reported slightly higher participation in gymnastics (15.2%) than men (12.1%).

Greater participation in PA, with the exception of cycling, was observed in towns with a population of over 20,000 than in those below 20,000 residents. In addition, a significantly higher share of men reported working in the garden (67.9%) than women (40%).

In general, former blue collar workers have lower participation in PA than former non-blue collar workers. The exception, however, is taking part in short walks around the house. Here, physical laborers demonstrate slightly higher participation (64.2%) compared to non-blue collar workers who do not typically undertake manual work (62.7%). In contrast, non-blue collar workers are more likely to take part in short walks (82%) than women blue collar workers. Among the manual worker group, a significantly higher share of men reported cycling (31% compared to 10%) and working in the garden (55.2% compared to 34.3%) (Table 1).

The most commonly-cited reason for engaging in PA, regardless of age, sex, place of residence or socio-professional status, was health, being given by 73.8% of men and 77.7% of women. In the 65–84 age group a significantly higher proportion of women (90.8%) declared that they undertake PA for health reasons than men (66.3%), but a much higher percentage of men (100%) declared such activity than women (50.,6%). (Table 2). Health was regarded as an important motivation for nearly 75% of respondents from towns of up to 20,000 inhabitants and manual laborers, as well as over 75% of respondents from towns with over 20,000 residents and non-blue collar workers. In cities over 20,000 residents, a significantly higher percentage of women (21.3%) declared undertaking PA “to relax” compared to men (4.6%). In contrast, a significantly higher share of men (16.5%) reported taking PA “on doctor’s advice” compared to women (2.9%). Regarding socio-professional status, a significantly higher proportion of men undertook PA “out of habit—I exercised when I was young” (18.9%) compared to women (4.6%) (Table 2).

The next most frequent motivations for undertaking PA declared by the respondents were “to relax” and “to kill time”. The former was declared by 25.2% of men and 31.7% of women, and the latter by 31.7% of men and 28.7% of women. In the 65–74 age group, a much higher percentage of women (27.0%) undertook PA “to relax” than men (18.1%). Similarly, in the 75–84 years old group, a significantly higher share of women (52.8%) reported undertaking PA “to kill time” than men (23.5%). However, in the over 85 age group, a significantly higher proportion of men (24%) reported taking part in PA “out of habit—I exercised when I was young” than women (0%).

Only fewer than 20% of men and 5% of women, a significant difference, reported undertaking PA “on doctor’s advice”. In addition, irrespective of age, a significantly higher proportion of men (17.7%) reported taking part in PA “on doctor’s advice” compared to women (5.3%).

**Figure 1:**
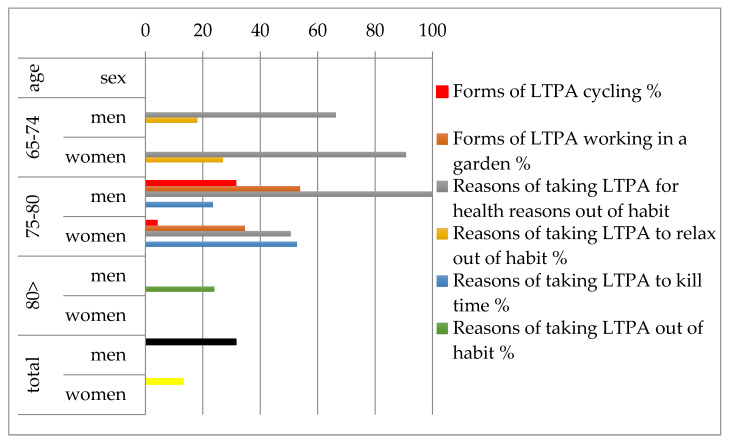
Statistically significant differences between men and women in LTPA forms and motives in terms of age.

Among the studied variables, the most statistically significant differences in the forms and motives of LTPA were recorded in terms of sex and age (Figure 1).

## 4. Discussion

The level of LTPA reported by seniors aged 65 and over from the Podlaskie Voivodeship is insufficient, and does not fulfil the WHO recommendations regarding the prophylaxis and prevention of chronic diseases [10,33]. This negative situation is also typical of other voivodships in Poland, including Wielkopolskie, Pomorskie and Małopolskie [41,42,43]. Furthermore, low levels of PA have also been reported by seniors throughout Poland [2,44,45], as well as in other European Union countries [46,47,48].

Furthermore, the participants indicate a decreasing participation with age. This trend can be attributed to the natural aging process. Older age groups are more prone to disability and fewer people can participate in PA. However, men tend to remain physically active longer with age, a trend that can be seen in all age groups. These results are consistent with those of the Eurobarometer study of PA among Europeans [46,47,48], and the Polish Central Statistical Office (GUS) [2,44,45].

Our finding that short walks are the dominant form of PA among seniors is consistent with the results of previous studies obtained for the Małopolskie, Wielkopolskie, Dolnośląskie and Pomorskie voivodeships [41,42,43]. However, long walks that enable the fulfillment of the WHO recommendations in the field of PA are the next declared form of PA. Long walks dominate among this type of recreational PA of the elderly in the nationwide PolSenior study for the whole of Poland [49,50,51]. Walking is the most common form of PA among older people. It is recommended for all age groups [52,53].

Considering that the Podlaskie voivodeship is a “green” region of Poland, with an above average afforestation compared to Poland as a whole, it is possible that participation in long walks may be associated with the forest cover of a given voivodeship: in greener areas, the residents can easily reach a natural area, or forests and parks with tree stands. A similar trend was observed in older men living in towns with more than 20,000 inhabitants in the Podlaskie, Dolnośląskie and Małopolskie voivodeships. In the Podlaskie Voivodeship, which is 30.9% afforested, 20.4% of older men report taking part in long walks, while in the Dolnośląskie Voivodeship, which is 29.8% afforested, 16.5% take part in long walks, and in the Małopolskie voivodship, 28.7% afforested, 11.1% of men take part in long walks [42,43,54]. These findings strongly suggest that among older men, participation in long walks increases with an increase in forest cover.

Otherwise, compared to the PolSenior findings [49,50,51], the seniors from Podlasie indicate similar levels of participation in other forms of LTPA, e.g., cycling or gymnastics, and a large number report working on the allotment or garden.

The observation that greater PA was observed among city dwellers than residents of small towns is also generally confirmed in the GUS study [2,44,45], with cycling being the exception. This difference may result from differences in the role played by a bicycle between villages, small towns and larger towns. In smaller towns, bicycles are more often used for short-distance transport, rather than a piece of equipment for LTPA, as is the tendency in larger towns [50]. In addition, higher levels of LTPA were reported by non-blue collar workers than blue collar workers, similarly to the national PolSenior findings [55,56].

Similarly to the seniors from Podlasie, health was also found to be the predominant motivation for LTPA in the Eurobarometer [46,47,48] and GUS studies [2,44,45], as well as various others [49,50,57,58]. Interestingly, our observation that seniors rarely undertake physical activity at the advice of a doctor has also been confirmed in previous studies [41,42,43]. Medical doctors should recommend PA more often to the elderly in order to consolidate their belief that PA is an essential part of a healthy human lifestyle and an important component in providing support to seniors. The positive role played by the physician as a specific authority in recommending and promoting PA has been highlighted previously [59]. In Poland, doctors tend to be highly regarded and recognised in society. As such, their recommendation that PA is an important component of a healthy lifestyle may serve to reverse the undesirable tendency toward a low participation in PA observed in Polish society.

Of course, among experts, professionals and researchers, PA is regarded as an essential part of a healthy lifestyle. They recommend that it should be undertaken regularly, at all stages of human development. It should be emphasized that, in accordance with the latest WHO recommendations, the greater recognition of the importance of PA has required the reshaping of the 2009 Human Nutrition Pyramid [60]. In 2016, this was developed into the Pyramid of Healthy Nutrition and Physical Activity, with PA at its base; hence, it should be undertaken regularly, preferably for a minimum of 30–45 min a day. The changes described above reflect progress in medical science, and take into account both recent scientific findings and the recommendations of recognized global expert centers [60].

As the level of PA is known among seniors to be lower compared to younger age groups, it is extremely important to promote its value among this older group.

Other researchers indicate that regular PA, also undertaken in “old age”, has a positive effect on the quality of life and successful aging [61], increases life expectancy [62] and reduces the risk of short life in the elderly [63].

As the authorities of Podlasie self-government units declare, the issue of the participation of seniors in the PA is of interest to them. PA was part of the Program for the elderly in Podlaskie voivodeship for the years 2016–2020, which was based on the Social Policy Strategy of the Podlaskie voivodeship until 2020. The health component of the Program makes specific reference to the weak social situation of seniors from Podlasie, which is related to, inter alia, their low participation in PA. The Program details activities intended to increase the awareness of seniors and their families about the role of PA in supporting human health, as well as initiatives involving preventive health care based on promoting PA [64].

The limitation of our research was the heterogeneity of the age group and the diversified size in the individual age groups studied.

We can also include as a limitation not taking into account data that would allow using the involvement in PA to calculate the metabolic equivalent of tasks (METs). In the future, we would like to include these parameters in our research to make it easier to compare our results with the results of other studies conducted in the field of PA research.

## 5. Conclusions

PA is known to have a significant influence on human health, and LTPA has been found to play a significant role in the prevention of chronic and civilization diseases. The level of LTPA reported by seniors aged 65 and over from the Podlaskie Voivodeship is insufficient and does not fulfil the WHO recommendations regarding the prophylaxis and prevention of chronic diseases. Due to the fact that the low level of PA is reported by seniors from Podlasie, and its decrease progresses with age, it seems necessary that voivodeship local self-government units not only promote PA among this age group, but also organize functionally beneficial forms of LTPA for seniors. Such an initiative will not only be beneficial for maintaining the physical fitness and health of seniors, it will also ensure their independence and improve the comfort and quality of their lives. There should be a greater effort by voivodeship local self-government units, doctors and experts to promote forms of LTPA that can be performed daily and independently at home by seniors, such as gymnastics, long walks, cycling and gardening, etc., with the aim of improving health. Unfortunately, in 2017–2018, as part of the National Health Program, when 308 health promotion programs were implemented, Podlaskie and Łódź voivodeships did not participate in this program. It seems that each voivodeship local government unit should use financial programs aimed at maintaining the physical fitness and health of seniors now and in the coming term. The main task of local government units is to implement such a policy, including health policy, with the best benefit for the inhabitants, without giving up funds from the state budget for health programs.

By involving successive groups of older people in regular PA, their physical fitness and health can be improved. Such activity can slow the disability that progresses with age, thus reducing the burden on state healthcare caused by expenditure on social and medical care for the elderly.

## Figures and Tables

**Table 1:** Forms of Leisure Time Physical Activity (LTPA) undertaken by the respondents at least several times a week or more, according to age, sex, place of residence and socio-professional status in %.

Factor	Categories	N	Sex	Short Walks Near the House %	Walking or Hiking Lasting a Few Hours %	Exercise: Gymnastics, Aerobics, etc. %	Cycling %	Working in a Garden %
Age years	65–74	28 35	Man Woman	60.6 76.1	18.4 17.1	14.1 23.1	34.6 19.2	61.0 40.8
	75–84	23 24	Man Woman	69.2 61.8	13.2 0.0	9.1 4.0	31.4 * 4.0	45.9 28.7
	>85	43 33	Man Woman	60.1 * 37.1	4.9 0.0	4.9 0.0	4.0 3.1	15.0 12.5
	Total	94 92	Man Woman	62.7 68.3	16.1 10.3	12.1 15.2	31.5 * 13.1	53.9 * 34.7
Size of place of residence number of residents	Up to 20.000	54 46 100	Man Woman Total	60.8 59.8 60.2	13.8 3.3 7.5	5.8 12.6 9.8	39.9 * 18.8 27.3	46.5 28.8 35.9
	More than 20.000	40 46 86	Man Woman Total	66.3 75.6 73.4	20.4 16.3 17.3	24.2 17.4 19.0	15.8 8.3 10.0	67.9 * 40.0 46.7
Professional/social status	Non-blue collar worker, farmer	63 59 122	Man Woman Total	64.2 57.3 60.0	11.1 7.0 8.6	6.9 6.4 6.6	31.0 * 10.8 18.5	55.2 * 34.3 42.3
	Blue collar worker	26 30 56	Man Woman Total	62.7 82.0 77.8	32.9 15.4 19.2	29.3 28.1 28.4	36.5 16.9 21.2	55.5 37.0 41.2

Legend: * statistically significant difference; *p* < 0.05.

**Table 2:** Reasons for taking up Leisure Time Physical Activity (LTPA) by the respondents with regard to age, sex, place of residence and socio-professional status in %.

Factor	Categories	N	Sex	“for Health Reasons” %	“to Relax” %	“to Kill Time” %	“out of Habit—I Exercised when I was Young” %	“on Doctor’s Advice” %
Age years	65–74	28 35	Man Woman	66.3 90.8 *	18.1 27.0 *	34.9 18.9	24.8 25.0	20.1 7.9
	75–84	23 24	Man Woman	100.0 * 50.6	49.7 43.5	23.5 52.8 *	9.6 11.8	12.5 0.0
	> 85	43 33	Man Woman	72.3 52.0	25.3 29.7	21.8 26.7	24.0 * 0.0	7.8 0.0
	Total	94 92	Man Woman	73.8 77.7	25.2 31.7	31.7 28.7	21.5 20.1	17.7 * 5.3
Size of place of residence number of resident	Up to 20,000	54 46 100	Man Woman Total	67.1 76.5 72.5	38.6 45.9 42.8	31.8 48.6 41.5	12.6 21.5 17.7	18.6 8.6 12.8
	More than 20,000	40 46 86	Man Woman Total	84.0 78.5 79.9	4.6 21.3 * 17.0	31.6 14.1 18.6	35.3 19.1 23.2	16.5 * 2.9 6.4
Professional/social status	Non-blue collar worker, farmer	63 59 122	Man Woman Total	64.9 77.9 72.2	26.1 39.1 33.4	36.2 37.4 36.9	18.9 * 4.6 10.9	19.5 7.7 12.9
	Blue collar worker	26 30 56	Man Woman Total	97.7 84.2 78.9	23.3 26.4 25.8	20.1 17.6 18.1	28.1 35.5 33.9	13.3 3.3 5.4

Legend: * statistically significant difference; *p* < 0.05.

## Data Availability

Data is provided on request due to restrictions (e.g., privacy or ethical).
